# Publisher Correction: Microbiota-induced T cell plasticity enables immune-mediated tumour control

**DOI:** 10.1038/s41586-026-10649-7

**Published:** 2026-05-18

**Authors:** Tariq A. Najar, Yuan Hao, Yuhan Hao, Gabriela Romero-Meza, Alexandra Dolynuk, Emma Almo, Dan R. Littman

**Affiliations:** 1 Department of Cell Biology, New York University School of Medicine, New York, NY USA; 2 Division of Advanced Research Technologies, Applied Bioinformatics Laboratories, New York University School of Medicine, New York, NY USA; 3 Perlmutter Cancer Center, New York University Langone Health, New York, NY USA; 4 Center for Genomics and Systems Biology, New York University, New York, NY USA; 5 New York Genome Center, New York, NY USA; 6 Howard Hughes Medical Institute, New York, NY USA

**Keywords:** Immunoediting, Immunosuppression

Correction to: *Nature* 10.1038/s41586-025-09913-z Published online 14 January 2026

In the version of this article initially published, there were typographical errors in the Fig. 2a timeline, where for α-PD-1 antibody injections, “4, 7, 10” appeared originally as “4, 7, 0,” while in Fig. 2c, the *x*-axis label now reading “TNF-BV650” appeared originally as “TN- BV650.” The figure is now amended in the HTML and PDF versions of the article.

